# Anti-tumor effects of pigment epithelium-derived factor (PEDF): implication for cancer therapy. A mini-review

**DOI:** 10.1186/s13046-015-0278-7

**Published:** 2016-01-08

**Authors:** Louiza Belkacemi, Shaun Xiaoliu Zhang

**Affiliations:** Department of Biology and Biochemistry, University of Houston, Houston, TX 77204 USA; Center for Nuclear Receptors and Cell Signaling, University of Houston, Houston, TX 77204 USA

**Keywords:** Anticancer agents, Cancer, Pigment epidermal derived factor, Phosphaplatins

## Abstract

Pigment epithelium-derived factor (PEDF) is a secreted glycoprotein and a non-inhibitory member of the serine protease inhibitor (serpin) family. It is widely expressed in human fetal and adult tissues but its expression decreases with age and in malignant tissues. The main anti-cancer activities of PEDF derive from its dual effects, either indirectly on the tumor microenvironment (indirect antitumor action) or directly on the tumor itself (direct antitumor influence). The indirect antitumor activities of PEDF were uncovered from the early findings that it stimulates retinoblastoma cell differentiation and that additionally it possesses anti-angiogenic, anti-tumorigenic and anti-metastatic properties. The mechanisms of its direct antitumor effect, however, have not been fully elucidated. This review highlights recent progress in our understanding of the multifunctional activities of PEDF and, in particular, its anti-cancer signaling mechanisms. Additionally, we discuss the possibility of using novel phosphaplatin compounds that can upregulate PEDF expression as a chemotherapy for cancer treatment.

## Background

Cancer, which remains the second-leading cause of death in the United States, accounts for nearly one in every four deaths. The American Cancer Society estimates that 569,490 Americans died of cancer in 2010 (www.cancer.org/docroot/stt/stt_0.asp), despite the significant advances in recent years in surgical resection, adjuvant chemotherapy, and radiotherapy. This situation highlights an urgent need for novel therapies, such as targeted therapy that focuses on particular pathways and mechanisms of tumor growth that are restricted to cancer. Pigment epidermal-derived factor (PEDF) is one of several cellular molecules that have been explored for use in targeted therapy.

PEDF (also known as EPC1) is a member of the serpin (serine protease inhibitors) superfamily of proteins, and is commonly expressed in normal tissues [1–5], and to a lesser extent, in tumorous tissues [6, 7]. PEDF exhibits a broad spectrum of activities, and the most prominent among these functions relies on its anti-angiogenic activity and neurotrophic effect [8]. Other activities include stimulation of inflammation [9], and tumorigenesis [10, 11]. Investigation of PEDF-knockout mice confirmed a crucial role for this factor as a regulator of the angiogenic functions [12]. Additional studies demonstrated that PEDF is a potent inhibitor of tumor angiogenesis [13], as this factor not only suppresses blood vessel endothelial cell proliferation and migration, but also enhances cell apoptosis [13, 14]. The anti-tumor effects of PEDF are particularly evident in tumors where PEDF exerts both an indirect impact on tumor angiogenesis and a direct effect on tumor cells. Such anti-tumor effects have been observed in cancers of different tissue origins, including prostatic [12, 15–17] ovarian [18, 19], and pancreatic carcinomas [20]; melanomas [21–23]; gliomas [24, 25]; and osteosarcoma [26–29]. Remarkably, PEDF displays the opposite effect in certain healthy tissues, especially neural cells. For instance, in cerebellum granule cells, PEDF protects against both natural and potassium-induced apoptosis via activation of pro-survival genes [30]. Moreover, in cultured retinal pericytes, PEDF prevents oxidative stress-induced apoptosis [31, 32]; whereas in vivo PEDF blocks light-induced apoptotic processes in photoreceptor cells [32, 33]. These studies and others indicate that PEDF plays a cardinal role in the physiology and pathophysiology of the human body, and that the mechanisms of action of this factor depend on whether PEDF targets a blood vessel or a tumor.

In this review, we discuss the anti-tumor activities of PEDF and focus on its dual role as an inhibitor (e.g., angiogenesis) and as an inducer of various vital biological processes that lead to the therapeutic effect via different mechanisms of action. Furthermore, we describe the potential use of a novel class of platinum-based compounds, the phosphaplatins, which upregulate PEDF expression, as a new treatment strategy for malignant tumors.

## Properties of PEDF and its receptors

(i) Structure of PEDF
  PEDF is a monomeric 50-kDa glycoprotein [34] encoded by the *SERPINF1* gene, which is localized on chromosome 17p13. The crystal structure of PEDF was successfully elucidated in 2001, revealing that PEDF has a tertiary structure similar to other members of the Serpin family. This structure contains three β-sheets and 10 α-helices [35, 36] as well as a usual reactive centre loop (RCL; residues 373–380) near the C-terminus [37, 38]. PEDF also exhibits an asymmetrical charge distribution across the whole protein, as one side of the protein is heavily basic and the other side is heavily acidic, leading to a polar 3-D structure. Some domain sites, such as those for binding to collagen, heparin, and hyaluronan, were mapped on the human PEDF using either protein chemistry or genetic engineering [39–42].
(ii) Amino acid composition and functional domains of PEDF
  At least four isoforms of secreted human and bovine PEDF have been detected [43]. PEDF is a polypeptide composed of 418 amino acids [44], most of which form secondary structures with the exception of the first 35 residues at the N-terminus (residues 1–35). The anti-angiogenic properties and neurotrophic activities localize to the N-terminal region of the polypeptide, whereas the C-terminal region interacts with the membrane receptor [45]. The distinct functional domains of PEDF are summarized in Table 1. The deduced amino acid sequence contains consensus sequences for N-linked glycosylation and several predicted sites for phosphorylation and O-linked glycosylation. The significance for some of these post-translational modifications is currently unclear.

**Table 1:** PEDF functional domains

Function	Peptide sites
Anti-angiogenesis	34-mer peptide region (residues 24–57)
Collagen binding (anti-angiogenesis)	Asp256,Asp258, Asp300 (negatively charged), Arg149, Lys166, Lys167 (positively charged). Asp255, Asp257 and Asp299 are critical to collagen-I-binding
Cell differentiation	44-mer peptide region (residues 58–101)
Heparin binding	Arg145, Lys146 and Arg148
Hyaluronan	Lys189, Lys191, Arg194 and Lys197 form a motif that is critical for hyaluronan binding (Becerra et al. [42])
Laminin binding	34-mer peptide region (residues 44–77) (Bernard et al. [46])
Phosphorylation	Ser24, Ser114, Ser227
Neurotrophy	44-mer peptide region (residues in humans 78–121)
Tumor cell apoptosis	34-mer peptide region (residues 24–57)
(iii) PEDF receptors identification and function
  For the last 15 years several research groups showed that PEDF distinct receptors, present in the plasma membranes of various cell types, can elicit different signals [46]. At least two of these PEDF receptors (PEDF-Rs) have been proposed by earlier studies. An 80-kDa PEDF receptor (PEDF-R^N^) with high affinity to the 44-mer PEDF peptide, is involved in neurotrophic activity, and a 60-kDa PEDF receptor (PEDF-R^A^) with high affinity to the 34-mer PEDF detected in plasma membranes of retina, retinoblastoma, and central nervous system [15, 36, 47–49]. The PEDF-R^N^ is a phospholipase and triglyceride lipase associated with triglyceride metabolism [36]. It is known in mice as adipose triglyceride lipase-ATGL, desnutrin, and patatin-like phospholipase domain containing protein-PNPLP2, iPLAζ, and in humans as transport secretion protein-2.2 (TTS-2.2)/independent phospholipase Aζ [50]. The result of the binding of PEDF to PNPLA2 stimulating a given molecular signaling pathway is still elusive. However, it has been suggested that the localization of PNPLA2 around the neural retina and the central nervous system may denote a neurotrophic role of this receptor upon activation of PEDF. Moreover, upon PEDF binding, the PEDF-R^N^ can potentially induce phospholipase A2 liberating fatty acids and lysophosphatidic acid from phospholipids [50–52], which could act as second messengers for signal transduction in neuronal cell development and survival, or possibly trigger anti-tumorigenic (e.g. apoptotic role of fatty acids omega-3 docosahexaenoic acid (DHA) in tumour cells [53]. The PEDF-R^A^ is a receptor for laminin as well [46]. The laminin receptor (LR)-interacting domain on PEDF is localized to a 34-aa peptide (aa 44–77); whereas the PEDF-interacting domain on LR is located to a 91-aa fragment (aa 120–210). A 25-mer peptide called P46 (aa 46–70), derived from the 34-mer peptide of PEDF, is the part that interacts with LR. The binding of the 25-mer PEDF region to the LR on endothelial cell triggers apoptosis, whereas angiogenesis, migration, tumour cell adhesion and proliferation are blocked. According to Bernard et al. [46], the binding of PEDF to the LR represents a novel signaling pathway for anti-angiogenic activities of PEDF. The investigators also suggest that PEDF binding to the LR could possibly stimulate multiple apoptotic pathways independent of the FAS/FASL death pathway including MAPK, JNK, and p38.
  Additionally, a group of extracellular proteins have been shown to influence angiostatic functions of PEDF. For example, PEDF binds to collagen I (Table 1), which might change the integrin–collagen I interaction, and affect endothelial cell adhesion and docking [41, 54] with subsequent negative effect on angiogenesis. Further, PEDF binding to collagen II [41], collagen III [41] or glycoaminoglycans [55] probably enable anti-angiogenic functions of PEDF as well.
  Recently, a new ~60-kDa PEDF binding protein has been purified from membrane extracts of bovine retina tissues, retinoblastoma cells [47, 48], and endothelial cells [56]. The protein matches ectopic F_1_-ATP synthase β-subunit and is being considered as another receptor for PEDF. PEDF interacts and inhibits endothelial and tumor cell surface F1-ATP synthase [56] and prevents the formation of ATP from ADP and inorganic phosphate by the enzyme. Since ATP and ADP have receptors on cell surfaces, they are likely to represent mediators of PEDF. A modification in ATP levels could negatively affect many cell biological processes including cell viability [56].
  Last year two other proteins; PLXDC1 (also called tumor endothelial marker 7 or TEM7; ~60-kDa) and PLXDC2 (also named TEM7-related or TEM7R, ~56-kDa) were identified as cell-surface receptors for PEDF. [2] These two membrane proteins have a large extracellular domain, a transmembrane domain, and an intracellular domain, and share about 50 % homology. Expression studies have uncovered overlapping between the two proteins. However distinctive tissue expression patterns of PLXDC1 [57, 58], and PLXDC2 [59–61] respond to PEDF. [2] Interestingly, each receptor can play separate roles in different cell types. For instance, exposing one type of cell from blood vessels to PEDF would normally kill them, but cells lacking PLXDC2 (but not those missing PLXDC1) could survive PEDF treatment. Cheng et al. [2] discovered that in the absence of PEDF, both PLXDC1 and PLXDC2 form complexes containing more than one copy of either receptor, but as soon as PEDF binds to the receptors, it caused these complexes to disassemble, leading to the activation of downstream signaling processes in the cell.
  All together these findings suggest that PEDF-Rs play a decisive role in cell biological functions. Thus, the search for new PEDF-Rs and the signaling pathways they stimulate after the binding of PEDF must continue. Comprehending PEDF receptors and their mechanisms will eventually lead to the development of new drugs that target these receptors to treat human diseases including cancer.

## PEDF secretion and function under normal conditions

PEDF was first purified from conditioned media from human retinal pigment epithelial (HRPE) cells [62]. Since then, this endogenously produced protein has been detected in many tissues, including eyes, liver, heart, and adipose tissue, implying that PEDF has diverse and significant biological activities. The reported functional association of PEDF with both organogenesis [12, 63, 64], and homeostatic maintenance of adult tissues/organs [12, 65–68] supports this notion. The physiological PEDF serum concentration in healthy people, however, remains controversial, as reports have demonstrated values ranging from 4 ng/ml to 15 μg/ml [69–74], depending on the method of measurement [4, 69]. Despite these inconsistencies, PEDF production clearly decreases with age and in some diseases, such as nephropathy [75, 76], suggesting the importance of this factor for maintaining homeostasis. In the particular case of cancer, contrary to expectations, a recent study of prostate cancer showed that although the expression of PEDF was only detected in few tumor cells by immunohistochemistry, PEDF level in the venous blood of patients with prostate cancer was significantly high [77]. The investigators speculated that the elevation of PEDF may represent a protective mechanism against the tumor, manifested by an anti-tumor effect in patients with aggressive prostate cancer [78, 79]. They proposed PEDF as a potential biomarker that would mirror the level of tumor aggressiveness and allow risk stratification of patients with prostate cancer. This example shows that further clarification of the clinical role of PEDF is essential for a better correlation of PEDF production in cancer patients with clinical and pathological parameters.

In summary, PEDF is a multifunctional protein and it possesses neurotrophic, neuroprotective, anti-angiogenic, and anti-cancer activities [34, 80–82]. In the following sections, we focus this review on the anti-cancer activities of PEDF.

## Effect of PEDF on tumors

**Fig. 1:**
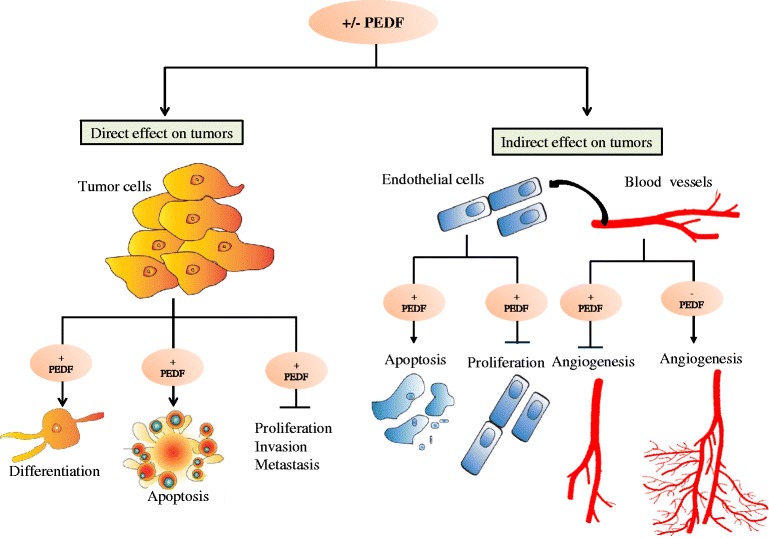
Modulation of the expression of PEDF affects tumor growth either (i) indirectly through the inhibition of angiogenesis, or directly via the prevention of cell proliferation, invasion and metastasis in concert with the promotion of cell apoptosis and/or differentiation. (−) PEDF means the absence of PEDF, (+) refers to the presence of PEDF, (→) means protein activation, and (—) means protein inhibition

One of the most interesting properties of PEDF is its anti-cancer effect. As detailed in the subsequent sections, this pluripotent protein can reduce tumor growth either indirectly through the inhibition of angiogenesis, or directly via the prevention of cell proliferation, invasion and metastasis in concert with the promotion of cell apoptosis and/or differentiation (Fig. 1).

A. Biological processes suppressed by PEDF
  (i) Angiogenesis
    A tumor is unable to grow to more than about 1 mm^3^ without the development of a new blood supply [83, 84]. Angiogenesis is a process that involves the growth of new blood vessels from pre-existing vessels [85]. This multistep process involves proliferation, migration, basement membrane degeneration, and new lumen formation, using the endothelial cells that line the inner surface of the blood vessels. This process is tightly regulated by both positive and negative signals [86, 87]. Among the positive factors, angiogenic vascular endothelial growth factor (VEGF) is one of the most highly expressed marker in tumors [88, 89]. Because of its varied anti-angiogenic functions, PEDF is considered one of the most significant negative regulatory factors.
    PEDF is a selective inhibitor of angiogenesis and targets only new vessel growth while sparing the pre-existing vasculature [90, 91]. Dawson et al. were the first to suggest a role for PEDF in angiogenesis [13]. They found that PEDF regulated blood vessel growth by creating a relaxed atmosphere for angiogenesis when oxygen was reduced (hypoxic conditions) such as in tumors, and an inhibitory environment when levels of oxygen were normal or high [13]. Doll et al. [12] also studied the role of PEDF in angiogenesis using PEDF knockdown mice. Their results showed that PEDF-deficient mice had significantly increased prostate stromal vasculature along with evident epithelial hyperplasia, suggesting a major role for PEDF in vascular/angiogenic functions. A notable characteristic of PEDF is its strong anti-angiogenic activity, as compared with other endogenous inhibitors of neovascularization, including thrombospondin, angiostatin or endostatin [13]. The exact molecular mechanism by which PEDF impedes angiogenesis is still not fully understood; however, PEDF-mediated anti-angiogenesis can result from enhanced gamma-secretase activity. This transmembrane cleaves single-pass transmembrane proteins at residues within the transmembrane domain, leading to the cleavage of the VEGF receptor-1 (VEGFR-1) transmembrane domain [92]. This action interferes with VEGF signaling, thereby inhibiting angiogenesis. In addition, as a major antagonist of VEGF, PEDF effectively blocks VEGF-driven angiogenesis and vascular permeability by regulating the proteolysis of VEGF [92]. The direct binding of PEDF to VEGFR-1 or VEGFR-2 promotes internalization and degradation of these receptors in VEGF-stimulated endothelial cells [93]. Thus, the interaction between PEDF and VEGFR-1 or VEGFR-2 represents a potential mechanism for the inhibition of angiogenesis. Hutchings et al. [14] have raised the possibility that PEDF may operate as a significant endogenous vasoactive substance, because PEDF exhibits synergistic action with VEGF in co-culture with endothelial cells, in conjunction with increased activation of the mitogen-activated protein kinases (MAPKs) [14]. Interestingly, PEDF has been found to induce the expression of pro-apoptotic FAS ligand (FASL) by nuclear factor kappa-light-chain-enhancer of activated B cells (NF-κB) in endothelial cells, and the regulation of NF-κB by PEDF is PEDF-R-dependent [94], it is therefore conceivable that PEDF may exert its anti-angiogenic activities through PEDF-R in endothelial cells.
    Degradative regulation of PEDF occurs under hypoxic conditions. Hypoxia is a primary physiological regulator of the angiogenic switch [95], and as such, low oxygen conditions downregulate PEDF expression [96]. This effect is due to the induction of matrix metalloproteinases (MMPs), which are an integral part of the extracellular matrix (ECM) enzymatic arsenal [97], and which proteolytically degrade PEDF. [97] In the presence of endogenous angiogenic inhibitors containing fragments or cryptic domains of large protein molecules [98–100] such as the plasminogen kringle domain 1–4 (also known as angiostatin) and the kringle 5 (K5) domain, however, PEDF expression is upregulated, and angiogenesis is hampered [23, 96]. Collectively, these data support the notion that PEDF is a powerful anti-angiogenic factor that negates VEGF activity and prevents tumor growth beyond certain size.
  (ii) Proliferation and invasion
    The ability of tumor cells to proliferate and invade healthy tissues is a fundamental characteristic of cancer. Cell proliferation results from the increase in cell numbers following cell division. PEDF is a negative regulator of cell proliferation. This protein inhibits the proliferation of various endothelial cells maintained in culture with fetal growth factors [14]. Likewise, exogenous PEDF delivered to cultures of endometrial cancer cells resulted in reduced cell proliferation [101]. In vivo, when B16-LS9 melanoma cells that had been engineered to overexpress PEDF were injected into the eye of C57BL/6 mice, the growth of the primary tumor was inhibited, and the number of metastases to the liver was decreased, confirming the anti-proliferative role of PEDF in these cells [102]. Konson et al. [103] showed that a triple phosphomimetic-altered PEDF is more efficient than wild-type PEDF in blocking neovascularization and tumor growth in vivo, and that this altered protein suppresses cultured endothelial cell proliferation much more effectively than wild-type PEDF. These findings are consistent with the role of PEDF as an inhibitor of cell proliferation. The exact mechanism by which PEDF reduces cell proliferation is still not well understood, but these actions may be associated with the levels of PEDF glycosylation, at least in endothelial cells. Consistent with this premise, Duh et al. [104] reported that secreted human recombinant PEDF (rPEDF) from human embryonic kidney cells has two species that vary in their carbohydrate composition at the N-glycosylation site, as well as in their efficacy of suppressing VEGF-induced proliferation of microvascular endothelial cells. Additional experiments, however, are required to ascertain the exact role of PEDF glycosylation in the mechanism of cell proliferation associated with tumor progression.
    When tumor cells and their progeny proliferate in an uncontrolled manner, they begin to disperse to other areas of the host (invasion) [105]. The role of PEDF in cell invasion has been highlighted by the finding that PEDF knockdown in poorly aggressive melanoma cell lines leads to increased invasion [106]. Similarly, PEDF interference significantly increased the migratory and invasive capability of normal melanocytes and enhanced their proliferative potential [106]. Conversely, exogenous PEDF delivered into endometrial cancer cells slowed their invasiveness [101]. PEDF attenuates cell invasion by disabling MMPs, which are key players in the events that underlie tumor cell invasion [107]. MMPs cleave cell surface receptors and degrade ECM proteins to induce invasion [108]. For example, membrane type-1 (MT1)-MMP facilitates the breakdown of the ECM to allow for aberrant tumor growth, and PEDF functions by preventing MT1-MMP distribution to the plasma membrane. The PEDF-dependent inhibition of MT1-MMP transport to the cell surface is mediated by a shift in the rat sarcoma homolog gene family member A (RhoA) and RAS-related C3 botulinum toxin substrate 1 (Rac1) balance [109]. RhoA and Rac1 play cardinal roles in cytoskeletal dynamics, cell movement, and various other common cellular functions [110]. Besides MMPs, PEDF modifies the expression of a number of genes that have been attributed in other studies to the malignant progression of human melanoma. Such genes include members of the SOX transcription factor family and genes that belong to the Notch (*NOTCH2NL* and *JAG1*) [111] or Wnt (*DKK1*, *FZD1*, and *LEF1*) [112, 113] pathways. Taken together, these studies validate the central role of PEDF in the prevention of tumor proliferation and invasion via various mechanisms, which are not completely understood and thus warrant further investigation.
  (vi) Metastasis
    Metastasis is one of the most significant problems associated with mortality in cancer patients [114, 115]. Metastasis occurs when cancer cells separate from the primary tumor mass and move to enter blood vessels. These traveling cells survive within the circulation by attaching to the endothelium of distant organs, entering the endothelial barrier, and establishing new tumor colonies, which are the primary cause of death. PEDF is a potent anti-metastatic protein that is lost in highly aggressive metastatic melanoma [106]. One good example is the orthotopic model, which was developed by Dass et al. [116]. The model demonstrates that PEDF has the greatest impact on metastases in mice injected with osteosarcoma lines, with a 70 % reduction in the development of pulmonary metastases and a 40 % decrease in primary tumor size versus untreated controls [116]. In addition, the expression of PEDF is inversely related to the metastatic potential and tumor grade of prostate adenocarcinoma [17], pancreatic adenocarcinoma [20], glioblastoma [25], hepatocellular carcinoma, and Wilm’s tumors [65]. The exact mechanism of action of PEDF on metastatic cancers is yet to be fully elucidated, although several reports shed some light on the numerous and complex signaling activities employed by PEDF to halt metastasis [23, 117]. For example, the VEGF/PEDF ratio has been proposed as an angiogenic switch in uveal melanoma metastasis [23]. Other studies identified MMPs as key players in the events that trigger tumor dissemination [118, 119]. For instance, in human chondrosarcoma cell lines that overexpress PEDF, MMP-14 levels were markedly low, and reduced trafficking of membrane-bound MMP-14 to the cell surface was observed, leading to decreased metastasis compared to matched controls [119]. Similar to invasion, this factor attenuates MT1-MMP function by preventing its distribution to the plasma membrane through RhoA inhibition and Rac1 activation [109]. In vivo, knockdown of Rac1 or overexpression of MT1-MMP was sufficient to reverse the inhibitory effect of PEDF on extravasation [109]. Reflecting the complexity of the phenotype that results from cancer metastasis, these findings suggest that several molecules interact with PEDF to attenuate tumor cell dissemination in various cancer types. As discussed in the next section, other biological processes, such as cell death and cell differentiation, are altered by PEDF overexpression as well, causing inhibition of tumor progression.

**Fig. 2:**
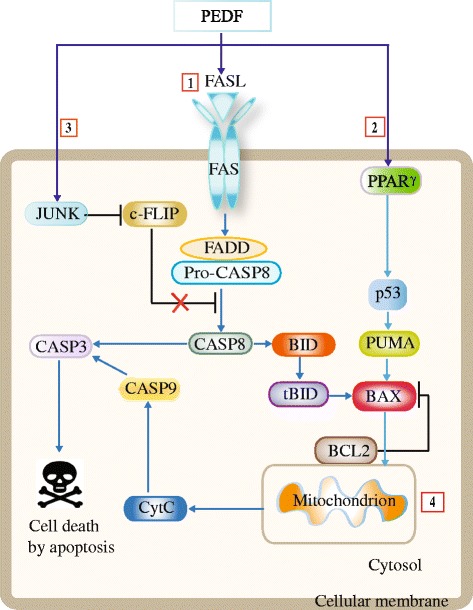
Different apoptotic pathways are modulated by PEDF. *1*. PEDF contributes to apoptosis in endothelial cells via the extrinsic FAS/FASL pathway [120] *2*. PEDF can sequentially induce the expression of PPARγ and p53 in endothelial cells. Stimulated p53 increases pro-apoptotic PUMA, inducing this protein to initiate the intrinsic pathway to apoptosis [130 *]* *3*. Also, PEDF mediates endothelial cell apoptosis through JNK activation, leading to cellular FLICE-like inhibitory protein (c-FLIP) blockade, which propels cells into a pro-apoptotic state [130]. *4*. PEDF can utilize the BCL2 family of proteins from the intrinsic pathway to stimulate apoptosis in tumors. Notably, activated caspase-8 stimulates apoptosis via two parallel cascades: it either directly cleaves and activates caspase-3, or it can cleave BID, a pro-apoptotic BCL2 family protein. tBID activation of BAX results in oligomerization of BAX on the cell membrane [146]. Anti-apoptotic BCL2 protein inhibits BAX oligomerization by sequestering tBID. The ratio of BAX/BCL2 may be more important than either promoters alone in determining apoptosis [147]. (→) means protein activation, and (—) means protein inhibition
    In addition, PEDF is engaged in the pathway involving c-Jun NH_2_-terminal kinase (JNK), a member of the MAPK family implicated in a range of biological processes associated with tumorigenesis [103]. It has been suggested that PEDF mediates endothelial cell apoptosis through JNK activation, leading to cellular FLICE-like inhibitory protein (c-FLIP) blockade, which propels cells into a pro-apoptotic state [130] (Fig. 2). The cFLIP protein is an intracellular inhibitor of caspase-8 activation that effectively prevents death signaling mediated by all known death receptors, including FAS, tumor necrosis factor receptor (TNF-R), and TNF-related apoptosis-inducing ligand receptors (TRAIL-Rs) [131]. PEDF also stimulates other well-known signaling molecules that contribute to cell apoptosis. One such molecule is NF-κB [132], which is stimulated by PEDF perhaps via the induction of *FASL*, a gene that contains NF-κB binding sites in its promoter region [133–137]. Collectively, these results underscore the idea that in both endothelial and tumor cells, PEDF acts on anti- and pro-apoptotic proteins from various regulatory mechanisms to promote apoptosis.
  (ii) Differentiation
    PEDF stimulation of retinoblastoma cell differentiation provided the first clue about PEDF-mediated reduction of harmful tumor phenotypes [37, 132, 138]. In these tumor cells, PEDF supports neurite development in concert with elevation of neuronal marker expression. Since then, Schwann cells, as well as other brain cells that naturally secrete PEDF, have been shown to cause neuroblastoma cell differentiation to yield a less malignant phenotype [139]. Remarkably, when Crawford et al. [139] injected rPEDF-expressing cells into neuroblastoma tumors in vivo, a pale region corresponding to confluent areas of spindle-shaped cells, which were characterized by bland nuclei with abundant cytoplasm, were observed. These spindled-shaped cells were relatively different from the more primitive neuroblasts observed within the tumor distant from the injection site, whereas the control cells were formed of undifferentiated neuroblast cells. These investigators also found that even treatment with lower PEDF levels induces discrete areas of tumor cell differentiation. The molecular mechanisms by which PEDF mediates tumor cell differentiation to a less malignant phenotype and protects normal neuronal cells likely involve a very complex system of regulation because the *PEDF* gene, which harbors a typical signal-peptide sequence, initiator methionine codon, and polyadenylation signal, and fits the size of the other members of the serpin superfamily (e.g., a1pha 1 anti-trypsin), is not homologous at the putative serpin reactive center [37]. This difference implies that PEDF may influence neuronal differentiation by a mechanism other than the common inhibition of serine protease activity. In other organs, such as the prostate, Filleur et al. [15] and Smith et al. [140] reported that PEDF contributes to prostate neuroendocrine differentiation via a feed-forward mechanism. The induction of neuroendocrine differentiation, however, may not be advantageous for patients with prostate cancer because PEDF expression is negatively regulated by testosterone [12], and high PEDF levels due to androgen ablation in cancer may cause expansion of the neuroendocrine component and influence both prostate development and prostate cancer progression [140]. Thus, the PEDF mediated neuroendocrine effect on cell differentiation warrants further investigation in order to gain a better understanding of the potential of PEDF use for the treatment of hormone-refractory diseases. Collectively, these findings suggest that PEDF has the ability to arrest tumor cell growth and induce differentiation to a less malignant phenotype while, perhaps simultaneously, protecting normal cells. Clearly, such a role requires a complex system of regulation, which may not always be compatible with the anti-cancer features of PEDF.

A. Biological processes promoted by PEDF
  (i) Apoptosis
    The assessment of anti-tumor effects of PEDF has uncovered apoptosis (physiological cell death) as a major anti-carcinogenic biological benefit of this factor. One of the fundamental mechanisms underlying the anti-angiogenesis activity of PEDF is its ability to selectively stimulate endothelial cell apoptosis in actively remodeling vessels [120, 121] consistent with the potency of PEDF as an anti-tumorigenic factor. In vitro, Guan et al. [16] observed that the overexpression of PEDF in prostate carcinoma cells was paralleled with elevated apoptosis in those cells compared to control cells. In vivo, PEDF exerts growth-suppressive and pro-apoptotic effects in lung cancer xenografts [122]. Apoptotic cells were also markedly increased in tumors derived from PEDF-overexpressing malignant melanoma cells, compared with control vector-transfected tumor cells [21]. Interestingly, when Konson et al. [123] compared the rate of apoptosis in PEDF-treated human breast adenocarcinoma (MDA-MB-231), human colon carcinoma (HCT116), and glioma (U87-MG) cell lines to that of PEDF-treated bovine aortic endothelial cells (BAEC) and human umbilical vein endothelial cells (HUVEC), they found that the apoptosis-inducing efficacy of PEDF in culture was stronger for endothelial cells, compared to tumor cells. Alternatively, cancer cells may be more resistant to PEDF action due to the higher basal activity of their survival pathways [123].
    PEDF stimulates apoptosis of endothelial and tumor cells through several pathways (Fig. 2). The extrinsic pathway, which is a cell surface death receptor-mediated pathway in which FAS is the most well-known player, and the intrinsic pathway, which is otherwise identified as the mitochondrial pathway because of a rise in mitochondrial permeability, are the best known. These two pathways have been extensively described (reviews [124–126]:). Therefore, our focus here will be on the interactions of these pathways with PEDF. The role of PEDF in apoptosis of endothelial cells via the FAS/FASL pathway has been demonstrated in elegant experiments by Volpert et al. [120]. These experiments revealed that the expression of pro-apoptotic FAS receptor is low in quiescent endothelial cells and blood vessels, but is upregulated by angiogenic factors, such as VEGF, resulting in sensitization of the stimulated cells to apoptosis by PEDF-generated pro-apoptotic FASL. This paradigm of cooperation between pro- and anti-angiogenic factors in the prevention of angiogenesis offers one possible explanation for the ability of PEDF to choose remodeling capillaries for destruction; however, the anti-angiogenic properties of PEDF are still evident when mice that are deficient for either FAS or FASL are treated with PEDF [127], implying that PEDF can also act independently of the FAS pathway to suppress angiogenesis. In tumors, PEDF stimulates apoptosis predominantly via the FASL/FAS pathway [122]; however, PEDF also utilizes the BCL2 family of proteins, which are part of the intrinsic pathway, for the induction of apoptosis. Zhang et al. [91] demonstrated that pro-apoptotic BAX protein was upregulated, and anti-apoptotic BCL2 was downregulated by PEDF in apoptotic glioma cell lines. Whereas these findings clearly validate a role for PEDF in the intrinsic pathway, whether PEDF utilizes any upstream regulators for BAX and BCL2 remains unclear. In fact, a pathway that incorporates both PEDF and the tumor suppressor p53, and includes BAX and other anti-apoptotic BCL2 family proteins has been described by Ho et al. [128]. These investigators specifically showed that when PEDF sequentially induced peroxisome proliferator-activated receptor gamma (PPARγ) and p53 expression in endothelial cells, a significant number of apoptotic cells were observed. The induction of p53 by PEDF, however, was abolished in the presence of PPARγ inhibitors. Furthermore, PEDF-mediated endothelial cell apoptosis was significantly reduced by p53 siRNA [128]. This implies that both proteins are pivotal regulators of PEDF-mediated apoptotic cell death. Stimulation of p53 can increase the pro-apoptotic protein p53 upregulated modulator of apoptosis (PUMA), inducing this protein to initiate the intrinsic pathway to apoptosis. The overexpression of PUMA leads to the upregulation of BAX, which undergoes conformation alterations and then translocates to the mitochondria with subsequent release of cytochrome *c* [129], resulting in the activation of caspases, and ultimately in the induction of apoptosis.

## Chemotherapeutic potential of PEDF

As an endogenous anti-tumor agent, PEDF has attracted wide attention. Immunohistochemical analyzes of PEDF expression in a variety of human tumor specimens and healthy tissues indicated that upregulated levels of PEDF expression were associated with a favorable prognosis, while decreased levels of PEDF were suggestive of a poorer prognosis [141]. Additionally, tissue microarray of matched primary and recurrent breast tumors after treatment with endocrine drug revealed that patients who had progressive disease, on average 93 months after endocrine therapy, had markedly lower levels of PEDF than those who showed a complete therapeutic response [141]. These findings has led to ongoing efforts focusing on the use of exogenous PEDF for cancer treatment. Two main approaches are considered for testing PEDF as a therapeutic agent. In the gene therapy route, PEDF was administered as an expressible form in a viral vector, and had demonstrated, as a proof of principle, that this form of therapy was effective [69, 142, 143]. Another route consisted of systemic administration of naked PEDF (free, unmodified) to tumors, which resulted in tumor regression caused by the discriminating effect of the protein on tumors and associated vasculature [65]. It is believed that with such drug delivery systems, enhanced drug pharmacokinetics and pharmacodynamics will be realized. However, such effort has met with limited success because of the necessity of continuous delivery of *PEDF* gene to sustain its expression in tumors. Moreover, as PEDF is expressed in healthy organs [144], the constant injection of this protein, with time, may affect not only the tumor but also the normal organ functions.

**Fig. 3:**
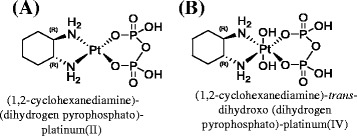
Structure of phosphaplatin compounds. (**a**) (1,2-cyclohexanediamine)-(dihydrogen pyrophosphato)-platinum(II), abbreviated as **RRD2**. (**b**) (1,2-cyclohexanediamine)-*trans*-dihydroxo (dihydrogen pyrophosphato)-platinum(IV), abbreviated as **RRD4**

A quite novel approach would consist of boosting PEDF expression via specific platinum-based chemotherapeutic phosphaplatin drugs. Phosphaplatins such as platinum[II] and platinum[IV] complexes (Fig. 3) that contain a pyrophosphate moiety exhibit excellent anti-tumor activities in a variety of cancers [145]. A leading compound in this class, RRD2 has recently been approved by the Food and Drug Administration as an investigational new drug, and is currently undergoing a phase I clinical trial (ClinicalTrials.gov Identifier: NCT02266745). The RRD2 significantly stimulates PEDF expression, and inhibits angiogenesis and cell migration of HUVECs (RN Bose patent: WO 2014130776 A1). Furthermore, these drugs stimulate PEDF expression in various tumor cells, including ovarian cells (RN Bose patent: WO 2014130776 A1). Current effort is devoted to developing drug delivery systems, such as controlled release nanoparticles that can potentially be used to deliver the drug to tumor tissues.

## Conclusion

Because PEDF secreted protein affects multiple physiological functions, it is an important determinant of optimal development, and ultimately, of lifelong health. PEDF has been established as an anti-cancer agent, utilizing several mechanisms for tumor inhibition. Although the diversity of PEDF activities may seem complex, these functions are consistent with its pleiotropic activities impacting both normal and malignant cells. Some of the molecular mechanisms of PEDF’s multifunctionality could be explained by responses to interactions with distinct cell surface receptors. Current investigations will undoubtedly clarify the details of PEDF signaling cascades and their biological importance. The importance of PEDF protein lies in its ability to inhibit tumor growth in several ways via anti-angiogenesis, anti-metastasis, induction of tumor cell differentiation and apoptosis. The distinctiveness of PEDF-mediated anti-angiogenesis stems from its ability to target only new vessel growth; however, this factor also is quite stable and nontoxic when injected systemically. Furthermore, the expression of PEDF can be induced by the nontoxic phosphaplatin drugs. With the completion of further studies, including those performed in our laboratory, demonstrating the anti-tumor effects of PEDF on different cancer types, the role of PEDF as a potential therapeutic agent is undeniably promising. As relatively little is known about the overall physiologic role of PEDF in the human body, further investigations are warranted prior to clinical studies of PEDF in cancer treatment.

## Consent for publication

Not applicable.
